# First-Principles Calculations of Two-Dimensional CdO/HfS2 Van der Waals Heterostructure: Direct Z-Scheme Photocatalytic Water Splitting

**DOI:** 10.3389/fchem.2022.879402

**Published:** 2022-04-07

**Authors:** Qiuhua Zhang, Kai Ren, Ruxing Zheng, Zhaoming Huang, Zongquan An, Zhen Cui

**Affiliations:** ^1^ School of Automobile and Aviation, Wuhu Institute of Technology, Wuhu, China; ^2^ School of Mechanical and Electronic Engineering, Nanjing Forestry University, Nanjing, China; ^3^ School of Mechanical Engineering, Wanjiang University of Technology, Ma’anshan, China; ^4^ School of Automation and Information Engineering, Xi’an University of Technology, Xi’an, China

**Keywords:** two-dimensional, CdO/HfS2 heterostructure, Z-scheme, photocatalyst, water splitting

## Abstract

Using two-dimensional (2D) heterostructure as photocatalyst for water splitting is a popular strategy for the generation of hydrogen. In this investigation, the first-principles calculations are explored to address the electronic performances of the 2D CdO/HfS_2_ heterostructure formed by van der Waals (vdW) forces. The CdO/HfS_2_ vdW heterostructure has a 1.19 eV indirect bandgap with type-II band alignment. Importantly, the CdO/HfS_2_ vdW heterostructure possesses an intrinsic Z-scheme photocatalytic characteristic for water splitting by obtaining decent band edge positions. CdO donates 0.017 electrons to the HfS_2_ layer in the heterostructure, inducing a potential drop to further separate the photogenerated electrons and holes across the interface. The CdO/HfS_2_ vdW heterostructure also has excellent optical absorption capacity, showing a promising role as a photocatalyst to decompose the water.

## Introduction

After the discovery of graphene in 2004 as a novel two-dimensional (2D) material (Geim and Novoselov, 2007), its outstanding thermal, electronic, and mechanical properties provide remarkable applications in many fields, also promoting the development of the other 2D materials (Cui et al., 2021a; Ren et al., 2021a; Zheng et al., 2021a; Cui et al., 2021b). Acting as popular layered material, transition-metal dichalcogenides (TMDs), expressed by XM_2_, where M represents transition-metal atom and X represents chalcogenide atom, is sandwiched by two chalcogenide atoms to form a sandwich structure (Hua Zhang et al., 2018). TMDs materials possess excellent electronic (Mak et al., 2010), thermal (Ren et al., 2022), thermoelectric (Wickramaratne et al., 2014), and optical (Ren et al., 2019a) performances. In recent studies, it has been proved that TMDs materials can be widely used in photocatalyst (Ren et al., 2020a), field-effect transistor (Yu et al., 2017), and photovoltaic devices (Gan et al., 2014). It is worth noting that the TMDs materials also can be prepared by an omnidirectional epitaxy (Xie et al., 2018), physical transport (Huang et al., 2014). Besides, the TMDs materials are also synthesized (Lu et al., 2017), suggesting novel photocatalytic properties (Zheng et al., 2021b; Lou et al., 2021; Zhu et al., 2021; Shao et al., 2022; Shen et al., 2022).

In recent years, using 2D materials as photocatalysts has aroused considerable focus (Wang et al., 2020a; Wang et al., 2020b; Wang et al., 2020c). The photogenerated electrons and holes in excited 2D semiconductors can quickly move to the material surface to participate in a redox reaction, which greatly shortened the photogenerated charge moving path, and a wider reaction area is also provided (Chen et al., 2010). However, the rapid recombination between the photogenerated electrons and holes hinders the reaction efficiency (Ren et al., 2019b). To solve this obstacle, many 2D heterostructures constructed intrinsic type-II band alignment have been investigated as photocatalysts because the lifetime of the photogenerated electrons and holes can be prolonged by separating into different layers. For example, the electronic and optical properties of AlN/Bp heterostructure present type-II band arrangement and have strong light absorption ability, which has great potential in the field of photocatalytic water decomposition (Yang et al., 2017). The experimental results demonstrate that under the condition of light, g-C_3_N_4_/Ca_2_Nb_2_TaO_10_ nanocomposite with a mass ratio of 80:20 has the highest hydrogen precipitation efficiency, which is more than 2.8 times that of single-layer g-C_3_N_4_ (Thaweesak et al., 2017). The nanorod array WO_3_/BiVO_4_ heterostructure was prepared by solvothermal technology. The experiments demonstrate that the photocatalytic performance of the heterostructure is significantly improved compared with the planar WO_3_/BiVO_4_ heterostructure. In particular, the IPCE value at 420 nm of the heterostructure film can be increased from 9.3% to 31% (Su et al., 2011). Similarly, the flower-like structure of CoNi_2_S_4_/Ni_3_S_2_ heterostructure was synthesized by the hydrothermal method, which shows that the electronic structure is optimized because of the high-intensity coupling between CoNi_2_S_4_ and Ni_3_S_2_, so as to improve the efficiency of photocatalytic water splitting (Dai et al., 2020). Furthermore, the Z-scheme photocatalyst is popular because of its extraordinary optical carrier moving path, which can provide more efficient photocatalytic performance. For example, the 2D C_7_N_6_/Sc_2_CCl_2_ heterostructure possesses ultrafast carrier recombination of about 0.74 ps, suggesting a strong redox capacity for water splitting (Meng et al., 2022). Z-scheme PtS_2_/arsenene heterostructure shows a novel high solar-to-hydrogen efficiency of about 49.32% (Ren et al., 2020b). The band bending mechanism in CdO/arsenene was addressed as a potential Z-scheme photocatalyst (Ren et al., 2021b).

More recently, the layered 2D CdO was prepared by the successive ionic layer adsorption and reaction method (Shameem et al., 2017) with outstanding electronic (Zhuang and Hennig, 2013), optical (Wang et al., 2020d), and electromagnetic properties (Zhao et al., 2019), which also can be tuned by the number of layers and stacking order (Hoat et al., 2020). At the same time, the external element doping for CdO can induce magnetic moment behavior (Chaurasiya and Dixit, 2019). In addition, 2D HfS_2_ was successfully prepared by the mechanical stripping method, which has attracted extensive attention from researchers (Kanazawa et al., 2016; Wang et al., 2017; Wang et al., 2019). HfS_2_ has a decent carrier mobility of 1,800 cm^2^ v^−1^ s^−1^ (Obeid et al., 2020). Importantly, HfS_2_ can be constructed into type-II heterostructure with other different 2D materials, showing an obvious quantum effect (Mattinen et al., 2019; Obeid et al., 2020). Considering the CdO and HfS_2_ monolayers share the same honeycomb structure and excellent physical and chemical properties, the CdO/HfS_2_ heterostructure is constructed in this report, using density functional calculations, the electronic properties of the CdO/HfS_2_ heterostructure are addressed by type-II band structure. Furthermore, the direct Z-scheme photocatalytic mechanism is also investigated for water splitting. Besides, the interfacial and optical performances of the CdO/HfS_2_ heterostructure are studied.

## Calculation Models and Methods

In this study, the simulations of the first-principles calculations were performed by the Vienna *ab initio* simulation package (VASP) based on density functional theory (DFT) (Kresse and Furthmüller, 1996a; Kresse and Furthmüller, 1996b). The generalized gradient approximation (GGA) was considered by the projector augmented wave potentials (PAW) using Perdew–Burke–Ernzerhof (PBE) functional for exchange-correlation functional (Perdew et al., 1996; Kresse and Joubert, 1999). The DFT-D3 method was used to describe the dispersion forces using Grimme (2006). Furthermore, the Heyd–Scuseria–Ernzerhof hybrid (HSE06) calculations are explored to obtain the electronic and optical characteristics (Heyd et al., 2003). In the first Brillouin zone, the energy cut-off was used by 550 eV, and the Monkhorst–Pack *k*-point grids were set as 17 × 17 × 1. In addition, 25 Å vacuum space was considered in this investigation. The force and energy were limited within 0.01 eV Å^−1^ and 0.01 meV, respectively, for convergence.

## Results and Discussion

**FIGURE 1:**
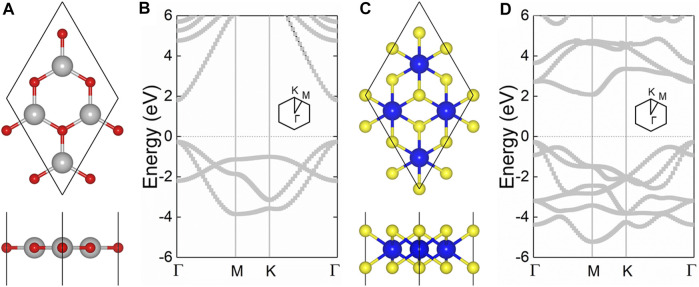
The geometric structures of the **(A)** CdO and **(B)** HfS_2_ monolayers; the band structures of the **(C)** CdO and **(D)** HfS_2_ monolayers; grey, red, blue, and yellow balls are Cd, O, Hf, and S atoms, the Fermi level is set to 0.

The hexagonal honeycomb structure of the CdO and HfS_2_ monolayers are optimized by the lattice parameters of 3.68 Å and 3.64 Å, respectively, demonstrated by Figures 1A,C. One can see that the CdO monolayer possesses a direct bandgap by the conduction band minimum (CBM) sharing the same point of *Γ* with the valence band maximum (VBM) in Figure 1B. While the HfS_2_ monolayer has an indirect bandgap with the CBM between the *Γ* and M, the VBM is found near the *Γ* point, as shown in Figure 1D. Besides, the HSE06 obtained bandgaps of the CdO and HfS_2_ monolayers are 2.07 and 2.05 eV, respectively. The results are in good agreement with the previous studies (Wang et al., 2020d; Obeid et al., 2020; Zhang and Ji, 2020).

**FIGURE 2:**
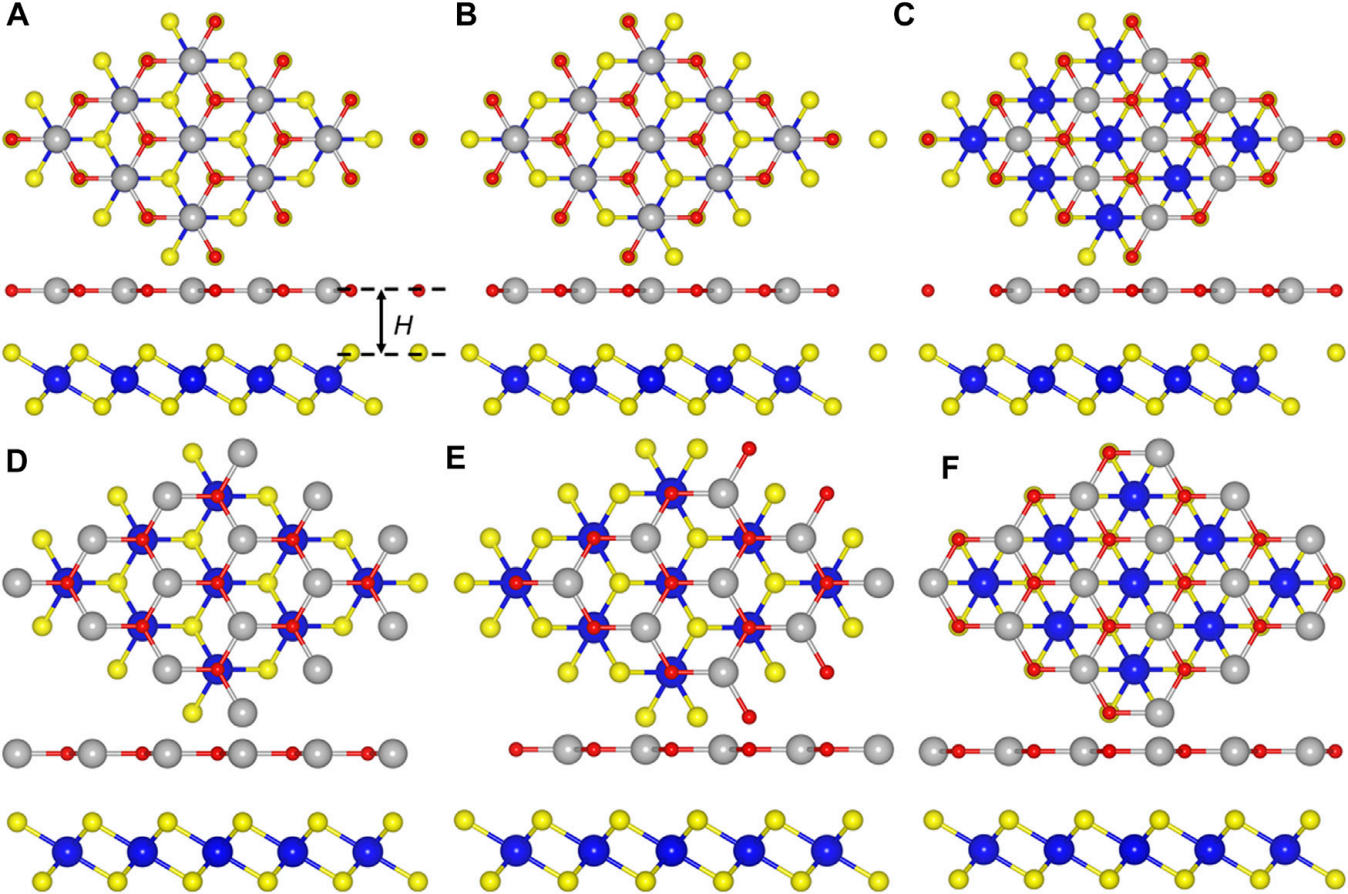
The top and side views of CdO/HfS_2_ heterostructure constructed by **(A)** CH-1, **(B)** CH-2, **(C)** CH-3, **(D)** CH-4, **(E)** CH-5, and **(F)** CH-6 configurations.

The CdO/HfS_2_ heterostructure is constructed in a vertical direction expressed by six different representative stacking configurations shown in Figure 2. We select the most stable stacking style by calculating the binding energy (*E*), which is obtained by *E* = (*E*_h_—*E*_CdO_—*E*_HfS2_)/*S*, where *E*_h_, *E*_CdO_, *E*_HfS2,_ and *S* represent the energy of the CdO/HfS_2_ heterostructure, original CdO, HfS_2_ monolayers and the area of the CdO/HfS_2_ heterostructure, respectively. Importantly, the obtained lowest binding energy is about −43.93 meV/Å^−2^ for CH-5 configuration, which is smaller than that in graphites of about −18 meV Å^−2^, revealing van der Waals (vdW) interactions between the interface of the heterostructure (Chen et al., 2013). Moreover, the following investigations of the CdO/HfS_2_ heterostructure are based on such a CH-5 configuration. Besides, the thickness of the interface of the CdO/HfS_2_ vdW heterostructure, explained by Figure 2A, is 2.86 Å, which is comparable with that of other vdW heterostructures such as ZnO/GaN (2.41 Å) (Ren et al., 2020c), BlueP/GeC, and BlueP/SiC (2.99 Å) heterostructures (Ren et al., 2019c) Table 1.

**TABLE 1:** The calculated binding energy (*E*, eV), bond length (*L*, Å), and the thickness of interface (*H*, Å) of the optimized CdO/HfS_2_ heterostructure constructed by different stacking styles.

	*E*	*L*_Hf–S_	*L*_Cd–O_	*H*
CH -1	−38.08	2.58	2.18	3.23
CH -2	−42.29	2.58	2.17	2.97
CH -3	−38.71	2.58	2.18	3.18
CH -4	−41.36	2.57	2.17	3.03
CH -5	−43.93	2.57	2.17	2.86
CH -6	−41.40	2.58	2.18	3.04

**FIGURE 3:**
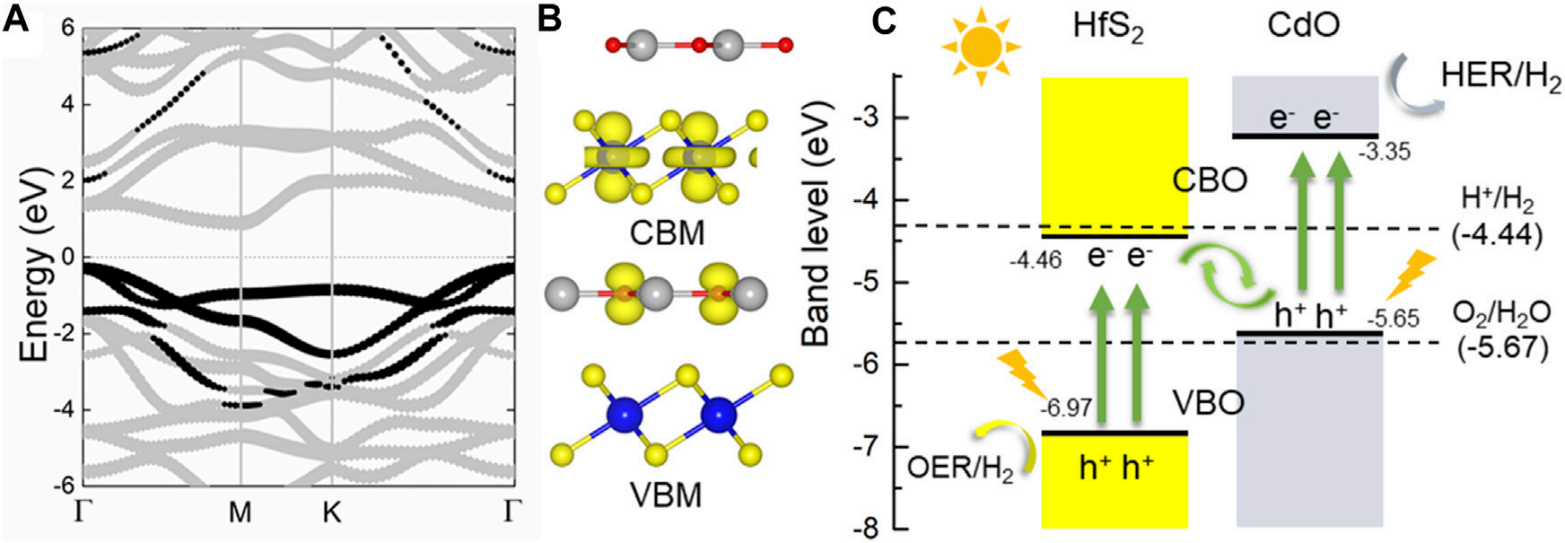
The **(A)** projected band structure, **(B)** band-decomposed charge densities, and **(C)** band alignment of the CdO/HfS_2_ vdW heterostructure compared with the oxidation and reduction of water splitting at pH 0; the Fermi level is set to 0.

Next, the electronic property of the CdO/HfS_2_ vdW heterostructure is explored by the projected band structure in Figure 3A with an indirect bandgap of 1.19 eV. The black and gray marks show the contribution of the band energy from CdO and HfS_2_ monolayers, respectively. Therefore, the CBM and the VBM of the CdO/HfS_2_ vdW heterostructure result from the HfS_2_ and CdO layers, respectively, further proved by the band-decomposed charge densities shown in Figure 3B, suggesting a type-II band structure in the heterostructure. This type-II band structure of the CdO/HfS_2_ vdW heterostructure can induce conduction band offset and valence band offset to further promote the migration of the photogenerated charges, revealed by Figure 3C. When the CdO/HfS_2_ vdW heterostructure is illuminated, the photogenerated electrons will move from VBM of the CdO (or HfS_2_) to the CBM, resulting in holes at VBM. Some photogenerated electrons (or holes) will be promoted from CBM (or VBM) of the CdO (or HfS_2_) to the CBM of the HfS_2_ (or CdO) by the conduction-band offset, CBO (or valence-band offset, VBO). Moreover, the remaining photogenerated electrons at the conduction band of the HfS_2_ and the photogenerated holes at the valence band of the CdO can make recombination at the interface of the CdO/HfS_2_ vdW heterostructure because of that specific band energy between the −4.44 and −5.67 eV at pH 0 (Ruiqi Zhang et al., 2018). In contrast, the band edge positions of the CBM and the VBM of the CdO and HfS_2_ are −3.35 and −6.97 eV, respectively, which are decent for the redox reaction for the water splitting (Xu et al., 2018). This extraordinary flow mode of the photogenerated charge suggests a Z-scheme photocatalytic mechanism in CdO/HfS_2_ vdW heterostructure, which is also reported by a MoSe_2_/HfS_2_ heterostructure (Wang et al., 2019).

**FIGURE 4:**
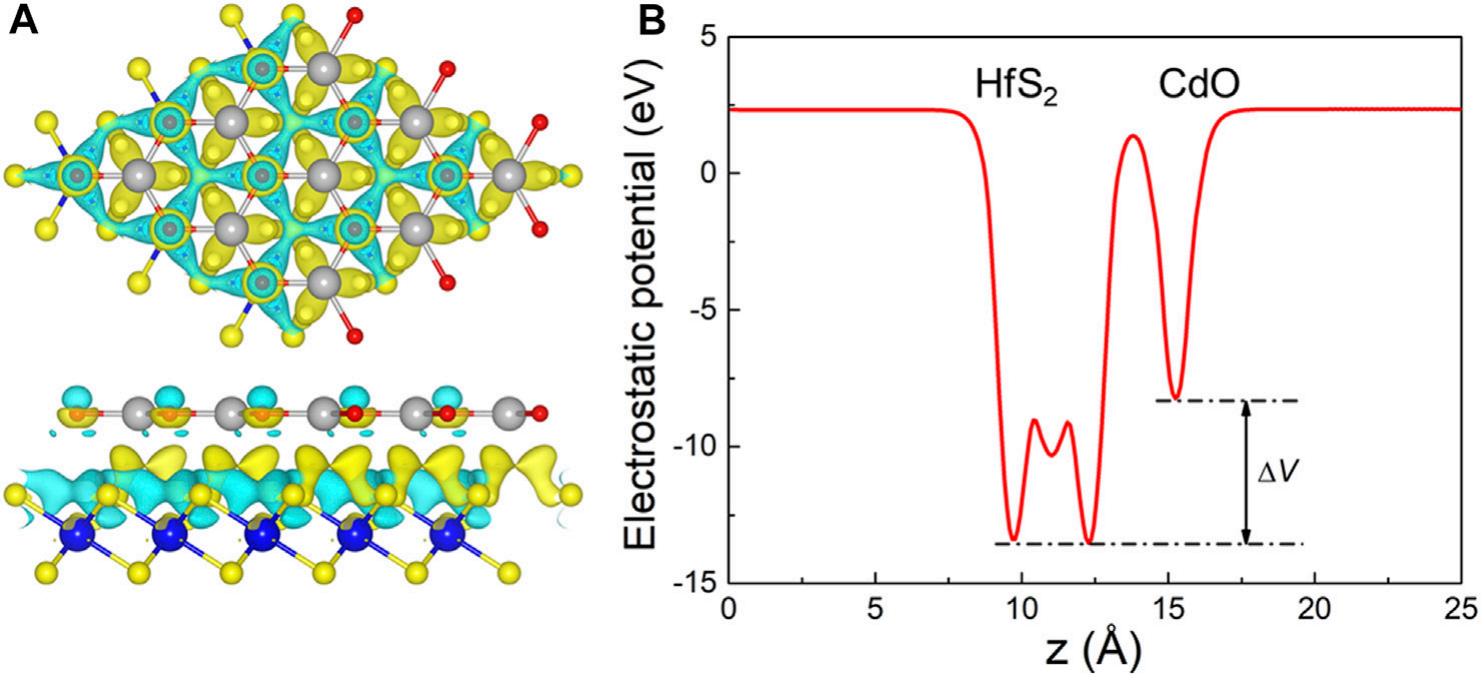
**(A)** The charge density difference and **(B)** potential drop at the interface of the CdO/HfS_2_ vdW heterostructure; the yellow and cyan regions mean the gaining and the loss of the electrons, respectively; the isosurface level is used as 10^−4^ |*e*|.

When the CdO and HfS_2_ layers contact, charge density difference (Δ*ρ*) occurs between the interface of the heterostructure, which is decided by Δ*ρ* = *ρ*_h_—*ρ*_CdO_—*ρ*_HfS2_, where *ρ*_h_, *ρ*_CdO_, and *s*_HfS2_ represent the charge density of the CdO/HfS_2_ heterostructure, original CdO, and HfS_2_ monolayers, respectively. The charge density difference of the CdO/HfS_2_ vdW heterostructure is addressed in Figure 4A, which shows that the electrons migrate from the CdO layer to the HfS_2_ layer. The charge density amount is investigated by Bader-charge analysis (Tang et al., 2009; Sanville et al., 2007) as 0.017 electrons. Besides, the potential drop (Δ*V*) of the CdO/HfS_2_ vdW heterostructure is also obtained in Figure 4B by 5.23 eV, which is larger than that of AlN/Zr_2_CO_2_ (0.66 eV) (Ren et al., 2021c) and Hf_2_CO_2_/GaN (3.75 eV) (Ren et al., 2021d). It is worth noting that this potential drop is also beneficial in promoting the separation of photogenerated charges (Wang et al., 2018).

**FIGURE 5:**
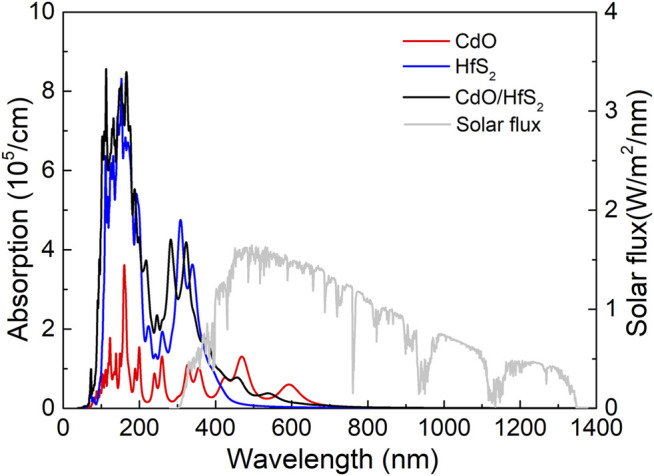
The optical absorption spectrum of the monolayered CdO, HfS_2_, and CdO/HfS_2_ vdW heterostructure calculated by the HSE06 method.

Light absorption capacity is essential performance as a photocatalyst for water splitting. The optical absorption properties of the CdO/HfS_2_ vdW heterostructure are calculated by $\alpha \left(\omega \right)=\frac{\sqrt{2}\omega }{c}{\left\{{\left[\varepsilon_{1}^{2}\left(\omega \right)+\varepsilon_{2}^{2}\left(\omega \right)\right]}^{1/2}-\varepsilon_{1}\left(\omega \right)\right\}}^{1/2}$ , where *α* is the absorption coefficient. The angular frequency and the speed of light are expressed by *ω* and *c*, respectively. The real and imaginary parts of the dielectric constant are represented by $\varepsilon_{1}\left(\omega \right)$ and $\varepsilon_{2}\left(\omega \right)$ , respectively. In Figure 5, the HSE06 obtained optical absorption spectra of the monolayered CdO, HfS_2_, and CdO/HfS_2_ vdW heterostructure are demonstrated by the absorption peaks of 3.56 × 10^5^ cm^−1^, 4.19 × 10^5^ cm^−1^, and 3.51 × 10^5^ cm^−1^ at the wavelength of 342, 323, and 351 nm, respectively, in the ultraviolet region. Importantly, the CdO/HfS_2_ vdW heterostructure possesses excellent visible light absorption capacity by the absorption peak of 7.21 × 10^4^ cm^−1^ locating at the wavelength of 465 nm, which is higher than other reported 2D heterostructures as photocatalyst, such as g-GaN/Mg(OH)_2_ (5.33 × 10^4^ cm^−1^) (Ren et al., 2019d) and ZnO/GaN (4.92 × 10^4^ cm^−1^) (Ren et al., 2020c). Besides, the CdO monolayer also shows a novel absorption peak of 6.01 × 10^4^ cm^−1^ in the visible light spectrum of 591 nm.

## Conclusion

In this work, the CdO/HfS_2_ is constructed by vdW interactions proved by first-principles calculations. The electronic properties of the CdO and HfS_2_ monolayers are calculated. In contrast, the CdO/HfS_2_ vdW heterostructure possesses a type-II band structure to prevent the recombination of the photogenerated charges. Furthermore, the decent band alignment of the CdO/HfS_2_ vdW heterostructure demonstrates a Z-scheme photocatalytic mechanism near the interface. Besides, the CdO/HfS_2_ vdW heterostructure shows pronounced visible light absorption performance. These results explain that the CdO/HfS_2_ vdW heterostructure can be used as a candidate for an excellent photocatalyst for water splitting.

## Data Availability

The raw data supporting the conclusion of this article will be made available by the authors without undue reservation.
